# Bacteria Mediate Oviposition by the Black Soldier Fly, *Hermetia illucens* (L.), (Diptera: Stratiomyidae)

**DOI:** 10.1038/srep02563

**Published:** 2013-09-02

**Authors:** Longyu Zheng, Tawni L. Crippen, Leslie Holmes, Baneshwar Singh, Meaghan L. Pimsler, M. Eric Benbow, Aaron M. Tarone, Scot Dowd, Ziniu Yu, Sherah L. Vanlaerhoven, Thomas K. Wood, Jeffery K. Tomberlin

**Affiliations:** 1 State Key Laboratory of Agricultural Microbiology, Huazhong Agricultural University, Wuhan, China; 2 Southern Plains Agricultural Research Center, Agricultural Research Service, United States Department of Agriculture, College Station, TX; 3 Department of Biology, University of Windsor, Windsor, Canada; 4 Department of Entomology, Texas A&M University, College Station, TX; 5 Department of Biology, University of Dayton, Dayton, OH; 6 Research and Testing Laboratory, Lubbock, TX; 7 Department of Chemical Engineering, Texas A&M University, College Station, TX; 8 Current address: Department of Biology, Queen's University, Kingston, Canada.; 9 Current address: Department of Forensic Science, Virginia Commonwealth University, Richmond, VA.

## Abstract

There can be substantial negative consequences for insects colonizing a resource in the presence of competitors. We hypothesized that bacteria, associated with an oviposition resource and the insect eggs deposited on that resource, serve as a mechanism regulating subsequent insect attraction, colonization, and potentially succession of insect species. We isolated and identified bacterial species associated with insects associated with vertebrate carrion and used these bacteria to measure their influence on the oviposition preference of adult black soldier flies which utilizes animal carcasses and is an important species in waste management and forensics. We also ascertained that utilizing a mixture of bacteria, rather than a single species, differentially influenced behavioral responses of the flies, as did bacterial concentration and the species of fly from which the bacteria originated. These studies provide insight into interkingdom interactions commonly occurring during decomposition, but not commonly studied.

Interactions between microbes and multicellular organisms are often challenging to characterize. No other place is this more apparent than in systems where there is competition for ephemeral resources. Janzen^1^ proposed that single-celled organisms on decomposing materials, such as seed, fruits and even carrion, function as more than simple nutrient recyclers. They are in fact members of the complex community competing for these resources, and through evolutionary time, have developed strategies for reducing competition with prokaryote and eukaryote consumers. However, it took 30 years before Janzen's concept was validated when Burkepile et al.^2^ reported that fish carrion contaminated with fewer bacteria were attractive to scavengers for a much longer period of time, and to a wider array of scavengers, than those with uninhibited bacterial fauna. These results indicated bacterial activity reduced competition with scavengers for the resource^2^. However, this effect did not apply to all competitors as some scavengers were actually more successful on the bacteria laden resource^2^.

Microbes have long been recognized for their functional importance in driving colonization of a resource by arthropods. Holdaway^3^ and Seddon^4^ proposed that ammonia produced by bacterial putrefaction on sheep stimulated oviposition by blow flies (Diptera: Calliphoridae). In comparison, gravid mosquitoes, *Aedes aegypti* (L.) (Diptera: Culicidae), must locate water sources that exhibit the appropriate environmental conditions for the development of their offspring^5^. A primary factor regulating attraction and colonization of these sites by female mosquitos is the associated microbial flora^6^, where it is the specific combination of 14 bacterial species responsible for the attraction^5^. Gravid house flies, *Musca domestica* L. (Diptera: Muscidae) evaluate volatiles produced by microbes on conspecific eggs to ensure synchronous larval development which allows for aggregative feeding and reduced likelihood of cannibalism^7^. Bacteria associated with these eggs also provided initial food resources^8^ and protection from pathogenic fungi on carrion^9^.

Recent advances in technology have expanded the tools available for the study of microbial ecology. Limitations existed in the ability to recover most community bacteria via conventional culture-based methods, thus providing gross under-estimates of microbial diversity in nature. Some anaerobic or microoxic bacteria require specific nutrients, or interactions with other organisms to grow and reproduce; factors that have made it problematic to replicate appropriate culture conditions in the laboratory. More recently, non-culture based techniques, such as molecular identification of bacteria via 16s rDNA, are being used to describe a more comprehensive bacterial community structure in different habitats and natural settings^10^. Consequently, more studies are using metagenomic methods to investigate microbial interactions with higher trophic levels^11^,^12^.

These tools can be directly applied to sustainable waste management and forensics. An organism that bridges these disciplines is the black soldier fly, *Hermetia illucens* L. (Diptera: Stratiomyidae). Adult black soldier flies lay eggs in a host of decomposing materials ranging from animal wastes^13^ to carrion^14^,^15^. Colonization of animal waste by black soldier flies often results in the exclusion of competing species, such as the house fly^16^. Past researchers speculated that this exclusion was due to the reduction in *Escherichia coli,* one of the primary food substrates of house flies located in the waste, by black soldier fly larvae^17^. The elimination or reduction of *E. coli* and other bacteria also is important to food safety to decrease transmission of pathogens to animals. In terms of forensics, this fly species frequently colonizes human remains and can be used to estimate a minimum postmortem interval^18^,^19^,^20^.

The black soldier fly is not the only species in these environments and often compete with a number of other insect species. The lesser mealworm, *Alphitobius diaperinus* (Panzer) (Coleoptera: Tenebrionidae) commonly occurs with the black soldier fly on decomposing matter in poultry operations. For decomposing carcasses, other insects such as the secondary screwworm, *Cochliomyia macellaria* (Fabr.) (Diptera: Calliphoridae) and the hairy maggot fly, *Chrysomya rufifacies* (Macquart) (Diptera: Calliphoridae), also are known to occur on the same resources as the black soldier fly. The occurrence of multiple species at the same ephemeral resource creates an environment for competition for resources, where direct and indirect interactions between insect species and microbes likely influence colonization patterns and local community structure; however there is little known about these interkingdom interactions.

Using bacteria isolated from black soldier fly food and identified through metagenomic 16S rDNA analyses, we ask if these bacteria attracted gravid females? We also determined if bacteria associated with black soldier fly eggs attract conspecific adult females. If there was increased attraction, we attempted to determine if it was associated with a single bacteria species or a more complex community. We hypothesized that bacteria found at a site used by the black soldier fly for egg deposition could influence insect attraction, colonization, and potentially succession. We demonstrated that bacteria from various life stages and species of insects significantly influenced oviposition preference by gravid black soldier fly females. Ultimately, such information could lead to the development of approaches to disrupt and manipulate the microbiota to produce communities engineered to repel pest insects, pathogen vectors, or attract beneficial insects.

## Results

### Conspecific eggs and substrate preference

Ephemeral resources played a role in attracting gravid black soldier fly females to oviposition sites (Table 2). The most significant (t = 3.36, df = 4, *P* = 0.028) increase in egg deposition in the presence of conspecific eggs occurred when no media was present. There was also an increase in oviposition in the presence of conspecific eggs when sterile substrate was present (t = 3.82, df = 4, *P* = 0.019). The number of eggs deposited between the different substrate treatments was similar (F = 1.45, df = 2, 6, *P* = 0.307) when conspecific eggs were already present. However, in the absence of conspecific eggs, there was less oviposition (F = 5.98, df = 2, 6, *P* = 0.037), primarily between the no substrate and non-sterile substrate treatments (Tukey's q = 4.66, *P* < 0.05).

### Sterility preference

There was a difference in egg deposition among sterility preference treatments (F = 28.39, df = 4, 12, *P* < 0.0001). Sterilization to remove microbes from eggs (T1) reduced oviposition preference by gravid females compared to the non-sterile (T2) (Tukey's q = 6.47, *P* < 0.05) and H_2_O rinsed (C1) egg (Tukey's q = 5.20, *P* < 0.05) treatments (Table 3). No eggs (C2) had the lowest level of attraction accounting for 7.4% of the eggs deposited. In contrast H_2_O rinsed (C1) (Tukey's q = 10.38, *P* < 0.05) or the Non-Sterile egg (T1) (Tukey's q = 11.65, *P* < 0.05) treatments had the highest levels of attraction, 34.8 and 39.2 respectively.

### Bacterial species preference

Black soldier fly preference for bacterial isolates was tested by oviposition responses (Table 4). All bacterial isolates tested were identified by Sanger sequencing using 16S rDNA sequence. Alignment of two universal bacterial primer sets allowed for the creation of a minimum 2× coverage consensus sequence of approximately 600 to 750 bp. Summary of the accession numbers, length of the sequences, and Ribosomal Database Project Naïve Baysian classifier identification can be found in Table 1. Of the isolates tested, *Ignatzschineria* sp. 2 and 3 from *C. macellaria* eggs, *Providencia* sp. from *Ch. rufifacies* 3^rd^ instar and *Acintobacter* sp. from *Alphitobius diaperinus* stimulated a significant response from the black soldier fly.

**Figure 1:**
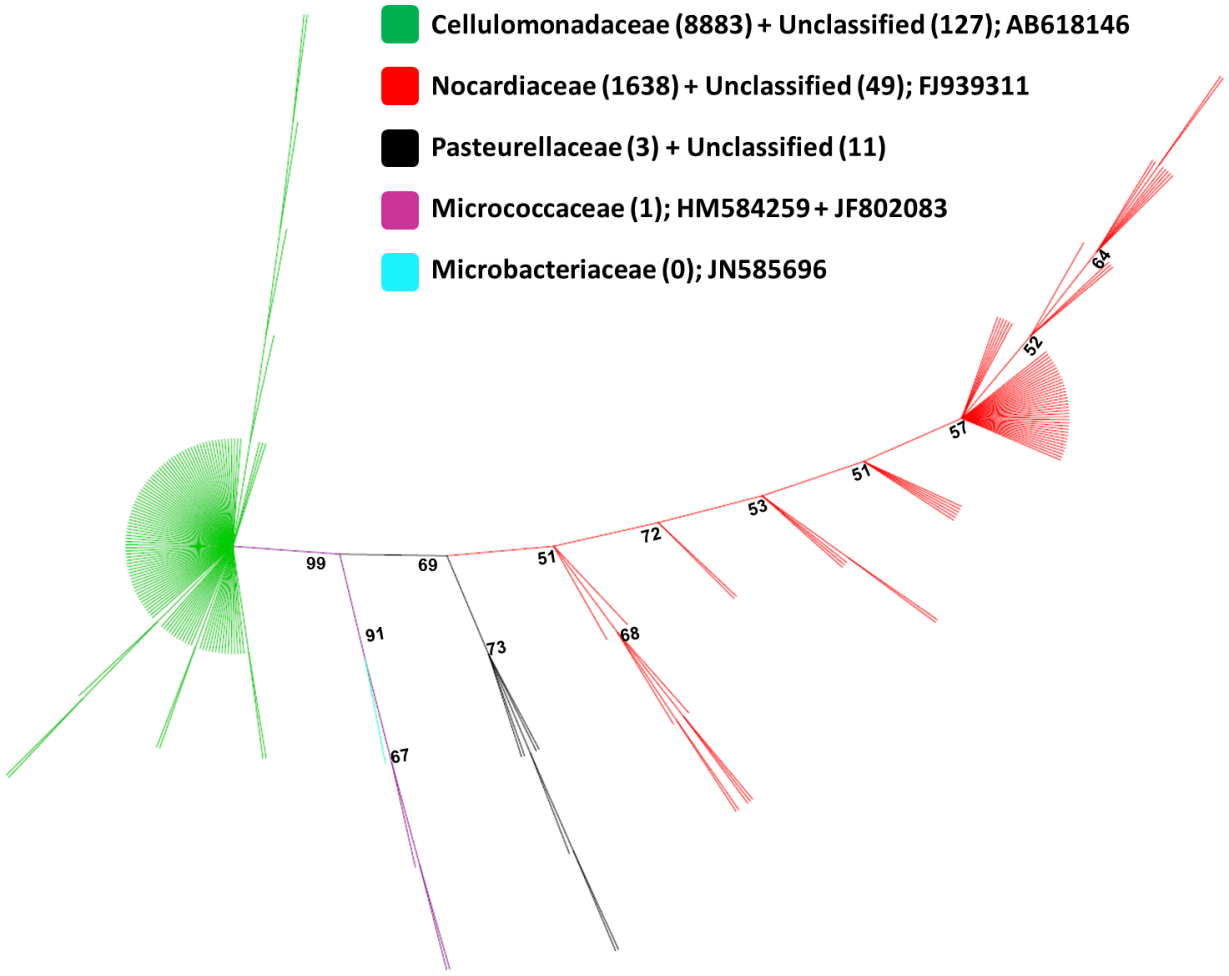
Unrooted neighbor-joining tree of 16S rRNA gene sequences associated with the bacterial mixture isolated from black soldier fly eggs. Values in the parentheses indicate total number of sequences obtained from pyrosequencing and assigned to a particular family or unknown group. GenBank accession numbers after the semicolon indicate those sequences that were downloaded from GenBank based on the best blast match with Sanger sequences. Sequences from both methods were used for construction of NJ tree. Numbers on the node indicate bootstrap values (bootstrap values for some terminal nodes are not shown). Branches are colored to indicate sequence assignment at family level.

In one instance, a mixture of gram positive bacteria was obtained from black soldier fly eggs (Mixture), which preferentially grew collectively and proved challenging to separate into individual isolates by culture methods. When this mix was tested for olfactory response as a collective it produced a significant oviposition response. Therefore, pyrosequencing was performed to determine its constituents. Out of total 10712 sequences, ranging in length from 250 bp to 518 bp (average length = 387 bp), 10702 (99.90%) were classified into order Actinomycetales (99.88%) and Pasteurelles (0.03%). Similarly, 10525 (98.25%) sequences were classified into 4 families Cellulomonadaceae (82.93%), Nocardiaceae (15.29%), Pasteurelleceae (0.03%), and Micrococcaceae (0.01%). At the genus level only 1623 (15.15%) of the sequence could be classified with ≥80% bootstrap support (default setting); *Gordonia* (99.51%), *Cellulomonas* (0.04%), *Gallibacterium* (0.03%) and *Micrococcus* (0.01%). Reducing the bootstrap cutoff to 50%, resulted in *Cellulomonas* identified as the most abundant genera (63.29%) followed by *Gordonia* (36%). In the Neighbor-joining (NJ) tree, all families (except Microbacteriaceae) were well supported monophyletic group (Fig. 1). Sequences that were unclassified at the family level using RDP classifier were clustered with the classified families with strong bootstrap supports (Fig. 1). Ultimately, four bacterial genera were isolated from the mix by subculturing using phenotypic characteristics and identified using capillary sequencing of the 16s rDNA as *Microbacterium*, *Cellulomonas*, *Gordonia* and two phenotypically different *Micrococcus*. These identifications complemented pyrosequencing results for the Mixture; therefore these isolates were tested individually for olfactory response. Only the *Gordonia* isolate, from the mixture, produced a significant response by the black soldier fly.

## Discussion

Ephemeral resources, such as carrion and plant material, represent valuable nutrients for a variety of species. The occurrence of carrion in an ecosystem is unpredictable and tends to degrade relatively fast. Further, competition between vertebrate scavengers and decomposers such as arthropods and microbes is intense^21^. Some arthropod species, such as the black soldier fly, have evolved to detect and locate these resources at the time of, or soon after, their demise^14^. Detection is predominately due to olfactory cues produced by these resources^22^,^23^,^24^ or conspecific offspring^25^.

We cultured aerobic bacteria from four insect species and isolated and characterized bacteria from 15 genera to test in this study. One of the bacteria was identified as *Empedobacter* sp. [JQ979474]; however Kämpfer, et al.^26^ suggest that the identification of accession #EU276091.1 may be *Wautersiella falsenii*. The Ribosomal Database Project naïve Baysian classifier identification of this isolate resulted in only a 71% bootstrap support, suggesting that identification to the genus level is tenuous. Three *Ignatzschineria* sp. were isolated from *C. macellaria* and sequence analysis determined only minor mismatches or gaps between the three sequences. *Ignatzschineria* sp. 1 [JQ979477] and 2 [JQ979478] aligned from the 4^th^ to the 661^st^ position with a single mismatch at the 53^rd^ position. *Ignatzschineria* sp. 3 [JQ979479] align beginning at the 61^st^ position, with a gap at the 169^th^ and 171^st^ position. This suggests that these isolates are all closely related. Similarity was also very high between the two *Micrococcus* isolates obtained from black soldier flies, Y [JQ979472] and W [JQ979473], with a single mismatch at the 626^th^ position.

Our study found that bacteria isolated from conspecifics on decomposing materials attract gravid black soldier flies, presumably by emission of volatiles. The same has been determined for other species including blow flies^27^,^28^. Bovine blood inoculated with bacteria isolated from wounds infested with *Cochliomyia hominivorax* (Coquerel) (Diptera: Calliphoridae) released volatiles attracting intraspecific adults^24^. House fly eggs are coated with microbes that release volatiles^7^. Concentrations thresholds of these volatiles dictate attraction and repulsion of conspecifics for oviposition behavior^7^. Colonization attempts when volatiles were above a threshold resulted in reduced survivorship of deposited eggs, while the opposite was determined for cohorts deposited with eggs emitting volatile concentrations below threshold levels^7^.

Oviposition responses of black soldier flies to bacteria isolated from competing arthropods were mixed. Significantly more eggs were laid in sites without the bacteria when black soldier flies were given a choice between sites with and without specific bacteria from *C. macellaria* (*Ignatzschineria* sp.) and *A. diaperinus* (*Acinetobacter* sp.), indicating repellency. This response by the flies is not unexpected as the species compete for the same resources. However in one instance, the black soldier flies responded to a *Providencia* sp. isolated from *C. rufifacies*. This unexpected response to bacteria from another species could be due to *C. rufifacies* being a newly introduced species to North America and consequently *H. illucens* had limited prior exposure to it. Secondly, *C. rufifacies* utilizes disparate resources, as they are predators on other blow fly species; therefore there could be less selection to avoid resources with *C. rufifacies* due to lack of competition.

A common approach when examining microbe-arthropod interactions is to isolate a single bacteria species and determine its impact on the arthropod of interest^24^. Such an approach is known to be limiting in terms of deciphering the true biological relevance of bacterial interactions with arthropod, as the behavior of the bacteria in isolation can be quite different than in the community mixtures typically encountered in the environment. We investigated the response of black soldier flies to a bacterial mixture isolated from conspecific eggs, as well as to several of its constituent species. We determined that the level of response was higher to the mixture than to any individual species. At a 50% bootstrap cutoff, a large component of the mixture were identified as genus *Cellulomonas*; a soil inhabiting Actinobacteria which have the ability to hydrolyze cellulose. The degradation of this major carbohydrate synthesized by plants represents an important part of the carbon cycle and this bacterium participates in the reduction of this biomass within the biosphere^29^,^30^. However, it was one of the lesser constituent species, *Gordonia* sp., which induced a significantly higher level of oviposition indicating its importance to the mixture and possibly to the ecology of the fly. Many Gordoniae can degrade xenobiotics, environmental pollutants, and other natural polymers; however, some are opportunistic pathogens^31^.

It is not surprising to have these bacteria present in such high numbers on black soldier fly eggs as these flies are known to colonize and develop efficiently on decomposing plant material^13^,^32^. On animal tissue, their development is greatly retarded with larvae needing an extra two weeks to complete development^33^. Such a response could be due to a lack of the carriage of appropriate bacteria to facilitate degradation of the different food source thus reducing required nutrient absorption. A potential solution could be taking a probiotic approach when using black soldier flies to reduce wastes other than plant materials. Inoculating a resource with the necessary bacteria prior to introducing black soldier flies could enhance their ability to recycle associated nutrients. Such an approach has been demonstrated in the past by inoculating poultry manure with *Bacillus subtilis* strains isolated from black soldier fly larvae, which enhanced larval weight by 30% and reduced development time up to 10%^34^.

Community level approaches to understanding microbial-insect interactions are integral for deciphering the “natural” mechanisms of ecosystem function. Researchers are now beginning to examine the interactions between bacterial communities, arthropods^5^,^35^,^36^ and hosts^37^. This should provide insight into these ecological interactions as it allows the bacteria to respond in a natural setting and results in a more accurate reflection of responses by arthropods. For example, oviposition response of the mosquitoes, *A. aegypti* and *Aedes albopictus* (Skuse) (Diptera: Culicidae) is influenced by the bacterial diversity and abundance associated decomposing leaf litter in artificial oviposition sites^5^. However, even in that study, a true appreciation of the bacterial diversity cannot be made due to limitations in techniques (i.e., polymerase chain reaction-denaturing gradient gel electrophoresis (DGGE)) utilized^5^. Unlike the Ponnusamy et al.^5^ study, we were able to use 454 sequencing to gain a greater appreciation of the bacterial diversity present in the microbial mixture isolated from black soldier fly eggs.

We were not able to conduct an in depth study of bacteria diversity associated with black soldier fly eggs due to financial limitations associated with 454 sequencing. However, with the trend towards reduced costs associated with high-throughput sequencing, collaborations between organismal and molecular ecologists are much more practical. This should allow future studies of insect-microbe interactions to explore a more full community level approach with microbes. In fact, future studies conducted with single microbe species, while valuable, should be explained within the context of the bacterial community as related to behavioral ecology.

## Methods

### Source of insects

Black soldier flies used in this study were obtained from a colony housed in a cage (1.8 m^3^ and 1.5 mm mesh screen) maintained year round in a greenhouse, outside the Forensic Laboratory for Investigative Entomological Sciences (FLIES) Facility located at Texas A&M University in College Station, TX, USA. The colony was established in the spring of 2009 from the eggs of a laboratory colony initiated at the Coastal Plain Experiment Station, University of Georgia, Tifton, GA, USA, which originated from material collected at a poultry facility in Bacon Co., GA, USA in 1998. Lesser mealworm colonies maintained at the Southern Plains Agricultural Research Center (SPARC, College station, TX, USA) were started from specimens isolated from a poultry farm located in Wake County, NC, USA. The SPARC colony has been in production since 2004^38^. Secondary screwworm and hairy maggot fly colonies were initiated from material collected from carrion located in the College Station, TX, USA vicinity during the summers of 2008 and 2009.

### Maintenance of the colony

Black soldier flies were reared according to the methods of Sheppard et al.^39^. Eggs were collected in a three layer, 3 × 5 cm corrugated cardboard block, held together with Elmer's® white glue with 3 × 4 mm flutes used as an oviposition substrate. Blocks were taped to the sides of a 22 × 22 × 10 cm square pan 5 cm above the oviposition substrate (moist-to-wet Gainesville diet)^40^ with the flutes perpendicular to the substrate. Collected eggs were labeled according to their date of oviposition to keep track of cohorts of the same generation. Briefly, eggs were held in 10 (L) × 10 (W) × 8 (D) cm plastic tubs at 27°C with ambient humidity until eclosion. The neonatal larvae were given approximately 200 g of Gainesville diet (5:3:2 hand mixture of wheat bran, alfalfa and corn meal, respectively, Producers Cooperative Association, Bryan, TX, USA)^13^. After 48 h, a fresh diet was added and the larvae were transferred into 40 (L) × 15 (W) × 10 (D) cm plastic pans as needed. The top of the pan was covered once per day with 2 cm of fresh diet (or as needed). Larvae were divided into new 50 (L) × 35 (W) × 12 (D) cm pans after approximately two weeks to maintain a density of approximately 2500 larvae per pan. When dispersing larvae accounted for 50% of the population in each pan, feeding was stopped and the remaining moist day-old food was allowed to dry to serve as a pupation substrate. Each pan of pupae was covered with white polyester organza fabric. Emerged adults were released into 1.5 m^3^ experiment cages held in the green house under natural light.

Lesser mealworm colonies were maintained using methods described in Crippen et al.^38^. Insects were housed in 15 (L) × 15 (W) × 30 (D) cm cages at 30°C on a 8:16 L:D cycle. Colonies were provided 1000 ml wheat bran (Morrison Milling Co., Denton, TX, USA) and 30 ml of fishmeal (Omega Protein, Inc., Hammond, LA, USA) as food. Deionized water was provided via a 36 cm^3^ sponge in each cage along with a 0.5-cm-thick slice of apple which was provided twice per week. Secondary screwworm and hairy maggot flies were maintained in separate cages using methods by Boatright and Tomberlin^41^. Adult flies of each species were housed in 30 cm^3^ Bioquip cages (Bioquip Products, Rancho Dominguez, CA, USA) in a 136LLVL Percival (Percival Scientific, Perry, IA, USA) growth chamber at ~27°C with 14:10 (L:D). Adult flies were provided a 50:50 sugar:powdered milk mixture *ad libitum*. Larvae were fed beef liver in 1.1 L styrene mosquito-breeding containers (Bioquip Products, Rancho Dominguez, CA, USA) held in the growth chamber previously described.

### Bacterial characterization by culture methods

Samples of approximately 0.2 g eggs, aged 24 hours, or ten 3^rd^ instar larvae were placed into a beaker with 2 ml DEPC dH_2_O and incubated at room temperature for 5 min, briefly shaken once per minute. The resulting supernatant was spread by 10-fold serial dilution onto Brain Heart Infusion (BHI) plates (Biolink Scientific, Austin, TX). Three replicates of each were incubated for 3 days at 26, 30 and 37°C, respectively. Individual colonies were selected phenotypically and sub-cultured for isolation onto trypic soy agar +5% sheep's blood plates (TSAB, BVA Scientific, San Antonio, TX, USA). The isolated bacteria were characterized by Analytical Profile Index (API) biotyping system (bioMerieux, Hazlewood, MO) and by 16S ribosomal DNA (16S rDNA) sequencing.

### DNA extraction, PCR amplification and sequencing

DNA template for Sanger sequencing was prepared by mixing 10 μl of an isolated colony in DEPC H_2_O and incubating at 100°C for 15 min followed by a brief centrifugation. The DNA for 454-pyrosequencing was prepared by modification of the protocol previously described in Zheng et al.^42^. Briefly, polymerase chain reaction (PCR) was performed from extracted DNA for the amplification of 16S rDNA using two independent sets of primers (short read primers (300 bp) forward: 5′- ACT TAA CCC AAC ATC TCA CGA, and reverse: 5′- AGG ATT AGA TAC CCT GGT AGT^43^, and long read primers (750 bp) forward: 5′- ACT CCT ACG GGA GGC AGC AG^44^, and reverse: 5′- AGG ATT AGA TAC CCT GGT AGT (Integrated DNA Technologies, Coralville, IA, USA) and PCR Master Mix (2×) (Fermentas, Glen Burnie, MD, USA). Since we were working with a wide variety of bacteria species, two different primer sets were used in two independent PCR's to assure good coverage and identification of a diversity of bacterial species. Thermal cycling for the short read primers consisted of an initial denaturation at 95°C for five minutes, then 30 cycles of denaturation at 94°C for one minute, annealing at 55°C for one minute, and extension at 72°C for one minute, and then a final extension at 72°C for 10 minutes. Thermal cycling for the long read primers consisted of an initial denaturation at 95°C for five minutes, then 40 cycles at denaturation at 94°C for 30 seconds, annealing at 50°C for one minute, and extension at 72°C for two minutes, and a final extension at 72°C for 10 minutes. The PCR products were purified using ExoSAP-IT (USB Corp., Cleveland, OH, USA) according to manufacturer's protocol and sequenced in both directions using standard ABI BigDye-terminator Cycle Sequencing (Applied Biosystems Inc., Carlsbad, CA, USA) protocols.

Sequences were edited for quality using 4Peaks (Mekentosj, Amsterdam, Netherlands)^45^ and aligned using MEGA version 5^46^ and a consensus sequence of a minimum 2× coverage was generated. The consensus sequences were submitted to GenBank, (see Table 1 for accession numbers). Consensus sequences were identified at genus level using Naïve Bayesian rRNA classifier version 2.2^47^ as implemented in Ribosomal Database Project (RDP) (http://rdp.cme.msu.edu/classifier/classifier.jsp) (accessed on October 4 2011) and at the species level using ≥97% sequence similarity cut-off in “blastn” algorithm of GenBank (NCBI, http://www.ncbi.nlm.nih.gov). When multiple species yielded identical scores, the sample was identified only to the genus level. *Gordonia* PCR products did not produce sequences sufficient to make a strong identification from the two universal bacterial primers, so a specific primer pair^48^ was used to confirm the identification of accession #JQ979470 (see Table 1).

### DNA extraction and pyrosequencing

One aliquot of 10 μl of the mixed bacterial culture was added in 500 μl Tris-EDTA (pH = 8), 50 μl 10% SDS, 3 μl proteinase K (20 mg/ml), 1.5 μl of lysozyme (50 mg/ml) and then incubated for 1 h with shaking (900 rpm) at 56°C in a water bath. After incubation, 100 μl NaCl (5 M) and 80 μl CTAB extraction solution (*Cat# C2190, TEKNOVA*) were added and samples were thoroughly mixed and incubated at 65°C for 10 minutes. Sequential extraction in a 1× volume was performed using phenol (pH = 8.0), phenol/chloroform/isoamyl alcohol (25:24:1), and chloroform/isoamyl alcohol (24:1) by centrifugation at 6000 × g for six minutes. The DNA was precipitated in 0.7 volume of isopropanol, washed twice in 70% ethanol, dissolved in nuclease free water, and quantified by spectrophotometry. Extracted DNA sent to Research and Testing Laboratory (http://www.researchandtesting.com/) for 16S rDNA 454-pyrosequecning using universal bacterial primer pair 28F (5′- GAGTTTGATCNTGGCTCAG) and 519R (5′- GTNTTACNGCGGCKGCTG) by bacterial tag-encoded FLX-Titanium pyrosequencing (bTEFAP) method^11^,^49^ in Genome Sequencer FLX System (Roche, Nutley, NJ, USA). All FLX related procedures were performed following Genome Sequencer FLX System manufacturers instructions (Roche, Nutley, NJ, USA).

### Pyrosequencing data analysis

Hierarchical classification of 10712 16S rDNA sequences were carried out according to the Bergey's bacterial taxonomy^50^ using Naïve Bayesian rRNA classifier version 2.2^47^ as implemented in Ribosomal Database Project (RDP) (http://rdp.cme.msu.edu/classifier/classifier.jsp) (accessed on October 04, 2011). Only sequences having ≥80% bootstrap support were considered classified at a particular hierarchical level.

To reduce computation load during phylogenetic analysis, almost identical reads (sequences with ≥98% similarity) (10505 sequences) were filtered using default parameters in cdhit-454^51^. Total 212 sequences (207 from pyrosequencing after cdhit-454 and 5 from Sanger sequencing) were aligned based on 16S rRNA secondary structure in Infernal aligner^52^,^53^, as implemented in the RDP under tool Aligner (http://rdp.cme.msu.edu/) (accessed on October 12, 2011). Because 16S ribosomal sequences obtained using pyrosequencing did not overlap with those obtained using Sanger sequencing in the multiple sequence alignment, we replaced Sanger sequences with those that were best match in NCBI BLAST search (AB618146, FJ939311, JN585696, HM584259, JF802083), but were longer in length. These sequences were realigned based on 16S rRNA secondary structure in Infernal aligner^52^,^53^, as implemented in the RDP under tool Aligner (http://rdp.cme.msu.edu/) (accessed on October 12, 2011). Hypervariable ambiguous regions were manually deleted from the multiple sequence alignment in MEGA5^46^. Evolutionary distances from 377 bp of aligned sequences were calculated by neighbor joining (NJ) method with the Kimura two-parameter correction^54^ for 1000 bootstrap replications in PAUP* v.4.0b10^55^. Calculated evolutionary distances were used for construction of unrooted NJ tree in PAUP* v.4.0b10^55^. All trees were edited using Archaeoptryx version 0.957 beta^56^.

### Experimental design for oviposition preference testing

Pans of pupae and newly emerged adults from the same generation were collected from the colony and released into three 1.5 m^3^ mesh cages over 5 consecutive days, providing a population of 5 cohorts of flies from the same generation, totaling approximately 2500 adults. On the fifth day, oviposition experiments began, having allotted 3 d for mating and 2 additional days for ovary development as described by Tomberlin and Sheppard^57^. Clear plastic tubs (22 (L) × 22 (W) × 10 (D) cm) sterilized with 80% ethanol were used as treatment containers. Corrugated cardboard blocks were sterilized under UV light for 30 minutes, treatments were applied (eggs, bacteria or nothing) and the blocks were taped one per side to the treatment containers. Oviposition preference was determined by measuring the mass of eggs oviposited into each flute of 5.0 (L) × 5.0 (W) × 0.2 (D) cm, triple layered, corrugated cardboard blocks. A female will only deposit one egg clutch and then will die^57^. Therefore, eggs deposit only represents clutches from individual females. Three replicates of each experiment were conducted for each of the three preference tests using approximately 2000 flies per cage with a 50:50 ♀:♂ ratio.

### Conspecific eggs and substrate preference

Two substrate diets were tested as long distance oviposition attractant: sterile, moist Gainesville diet and non-sterile, moist Gainesville diet. The non-sterile substrate consisted of 250 g of fresh Gainesville diet moistened with 700 ml _d_H_2_O and the sterile substrate consisted of 250 g of fresh Gainesville diet which was autoclaved, then moistened with 700 ml of autoclaved _d_H_2_O. The control consisted of an identical container with no diet substrate. The short distance oviposition attractant tested was the presence or absence of black soldier fly eggs which was tested with each of the two diets. Cardboard blocks were seeded with approximately 0.04 g of conspecific 1-d-old black soldier fly eggs divided evenly between two cardboard flutes. Of the four cardboard blocks, two containing eggs and two, taped on opposite sides, containing no eggs.

Oviposition preference site was tested for 60 minutes following the first active oviposition observed (t = 0). Three replicates in each cage with one cohort of flies were run consecutively in one day, rotating the position of each treatment and control within the cages. These replicates were run daily over 3 consecutive days, rotating treatments and controls between cages, totaling 9 replicates (N = 9). Oviposited eggs were dissected out of the cardboard flutes and weighed. The percent oviposition per treatment per substrate was determined gravimetrically.

### Sterility preference

Approximately 0.04 g of 1-d-old black soldier fly eggs were used for each of two treatments, Sporgon® sterilized 1-day-old eggs (T1) and non-sterile 1-day-old eggs (T2), and two controls, non-sterile 1-day-old eggs washed with sterile water (C1) and no eggs (C2). The T1 eggs were sterilized by placement into 1 ml of Sporgon® (Decon Laboratories, Inc., King of Prussia, PA) for one minute, with occasional gentle shaking, then removed from the solution and allowed to stand at room temperature for two minutes, followed by a rinse with two 1 ml aliquots of sterile _d_H_2_O and air dried for 30 minutes. T2 non-sterilized eggs were not treated with Sporgon® prior to use. C1 control eggs were rinsed twice with 1 ml sterile _d_H_2_O and air dried for 30 minutes prior to use. Using sterile forceps, the eggs were then evenly divided between two cardboard flutes of UV sterilized cardboard blocks.

For this experiment, four cardboard blocks (T1, T2, C1 and C2) were taped 3 cm above the Gainesville diet substrate. Oviposition preference was tested for 60 minutes after t = 0. Three replicates were run simultaneously in three cages. During each day, treatments were replaced in the cages at four consecutive times. With each replacement, treatments were rotated within and between cages for a total of twelve replicates per day. Eggs deposited were dissected out of the cardboard flutes and weighed. Percent oviposition per treatment per substrate was determined.

### Bacterial preference

**Figure 2:**
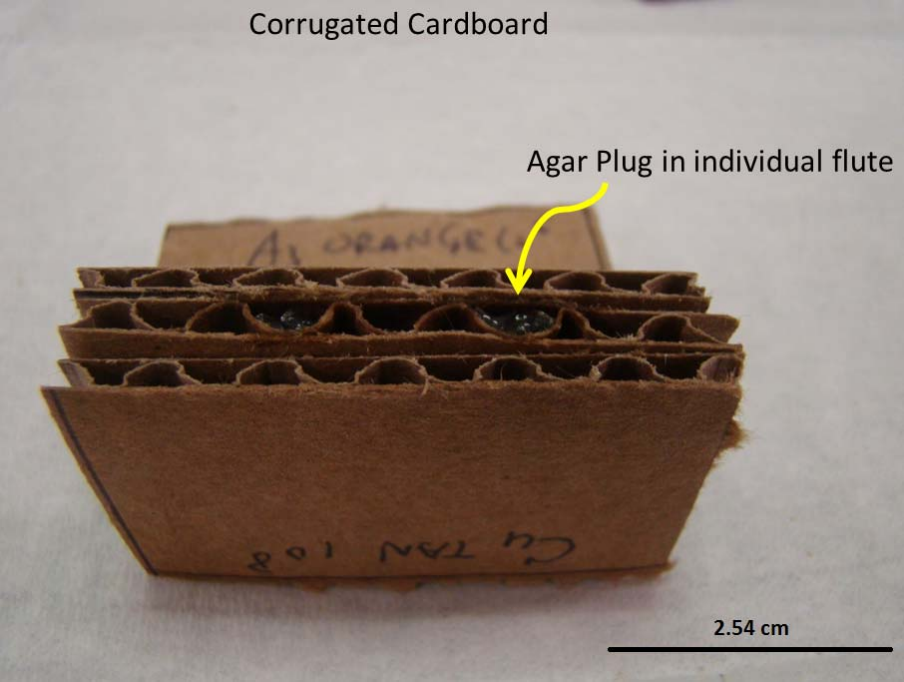
Olfactory test block showing 2 × 5 cm triple layered corrugated cardboard block into which a 0.5 g sterile agar plug could be added to the flute. An aliquot of 8 μl bacteria in PBS at the desired concentration could then be added onto the top of plug.

**Figure 3:**
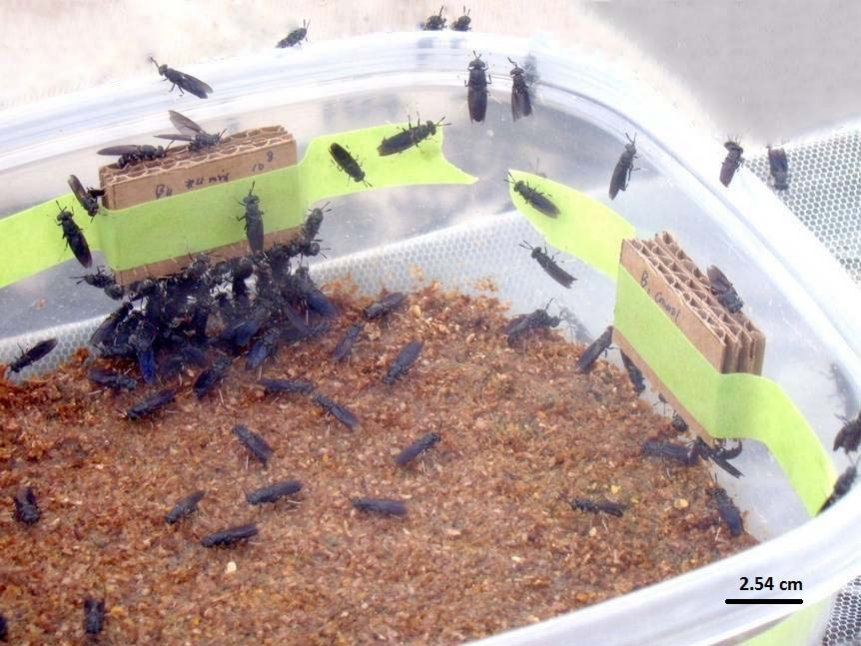
Olfactory test showing triple layered, corrugated cardboard block, taped one per side in 22 × 22 × 10 cm container, at 3 cm above the diet. This design was used to present bacteria (in the flutes) to black soldier flies and test oviposition site preference. In this view 10^8^ cfu/ml of the bacterial mix is in the left block and control (agar only) is in the right block.

An aliquot of 8 μl of 18 bacteria strains and one bacterial mixture, at 10^4^, 10^6^, or 10^8^ colony-forming units (cfu)/ml or a PBS (control) was added to the top of two plugs of BHI agar (approximately 0.5 g) sterilely inserted in two flutes each of a UV sterilized cardboard block (Fig. 2). One bacterial concentration was used per treatment block. Three treatment blocks (one treatment bacteria at each of three different concentrations) and one control block (consisting of sterile agar) were taped, one block per side, on a clear 22 (W) × 22 (L) × 10 (D) cm container, 3 cm above the moist-to-wet Gainesville diet to test oviposition site preference (Fig. 3). These replicates were run over three consecutive days at the same time each day, in four replicate cages (N = 12). In each cage the treatment faced a different direction (N, S, E, or W); which was rotated within each cage, each day. During the experiment the naturally lit green-house was kept at a constant temperature of 26°C. Eggs were removed from the cardboard and weighed as previously described. Percent oviposition per treatment was determined.

### Statistical analyses

Oviposition differences for conspecific eggs and sterile/non-sterile substrates were analyzed using a student's t-test while the effect of egg sterilization was tested using one-way ANOVA Tukey's post-tests. Both analyses were performed after arc-sine square root transformation of percentages.

Oviposition preference for different bacterial strains was analyzed using both parametric (one-way ANOVA with Tukey's post-tests) and non-parametric (Kruskal-Wallis with Dunn's post-tests) approaches to allow for appropriate statistical interpretation. This was done to balance interpretation and reduce the risk of Type II error. We preferred the more powerful one-way ANOVA where the assumptions of normality and homogeneity of variances were violated even after arc-sine square root transformation of percentages; however, we also used the Kruskal-Wallis rank test that is less powerful but does not have data structure assumptions. Significance was set at *P* < 0.05.

## Author Contributions

L.Z., L.H., T.L.C. and J.K.T. conducted the behavioral research. B.S. and M.L.P. conducted the molecular research and associated analyses. S.D. conducted the sequencing. M.E.B. conducted the statistical analyses on the behavioral data. T.L.C., J.K.T., A.M.T., Z.Y., S.L.V. and T.K.W. provided financial support. All authors assisted with drafting the manuscript for submission.

## Figures and Tables

**Table 1:** Sanger sequencing results of 16S rDNA of isolated bacterial strains. Lists insect species from which initial bacterial isolate was obtained (source) and GenBank (http://www.ncbi.nlm.nih.gov/genbank/) accession numbers of newly submitted sequences

Source	Accession Number	Length*	RDP ID†	Bootstrap support (%) ‡
*Alphitobius diaperinus*	JQ979481	733	*Acinetobacter* sp.	100
*Chrysomya rufifacies*	JQ979484	737	*Klebsiella* sp.	100
	JQ979485	725	*Morganella* sp.	100
	JQ979486	732	*Proteus* sp.	100
	JQ979480	677	*Providencia sp.*	100
*Cochliomyia macellaria*	JQ979483	723	*Hafnia* sp.	100
	JQ979477	600	*Ignatzschineria* sp.	100
	JQ979478	656	*Ignatzschineria* sp.	100
	JQ979479	662	*Ignatzschineria* sp.	100
*Hermetia illucens*	JQ979475	688	*Bacillus* sp.	100
	JQ979469	682	*Cellulomonas* sp	100
	JQ979474	615	*Empedobacter* sp.	71
	JQ979482	663	*Enterobacter* sp.	97
	JQ979470	718	*Gordonia* sp.	100
	JQ979476	691	*Kurthia* sp.	99
	JQ979471	666	*Microbacterium* sp.	100
	JQ979472	664	*Micrococcus* sp.	100
	JQ979473	666	*Micrococcus* sp.	100

*Number of base pairs of the consensus sequence represented by a minimum of 2× sequence coverage.

^†^Taxonomic identification made for that sequence by the Ribosomal Database Project naive Bayesian classifier.

^‡^Bootstrap percentage support for that identification.

**Table 2:** Percent ± SE (n = 9 experiments) black soldier fly, *Hermetia illucens*, egg deposition in oviposition sites with and without conspecific eggs nested within different substrate treatments

	Substrate Treatments		
	No Substrate	Non-sterile Substrate	Sterile Substrate†
Eggs	79.3 ± 11.5^a,c^	52.2 ± 10.7^a,c^	60.1 ± 8.3^a,c^
No Eggs	9.5 ± 6.0^b,c^	36.6 ± 9.6^a,d^	28.9 ± 5.1^b,d^

^†^Diet sterilized by autoclaving.

^a–b^Sample groups (Eggs and No Eggs) with the same letter (a or b) are not significantly different (*P* < 0.05) as compared within Substrate Treatment.

^c–d^Substrate treatments (No substrate, non-sterile substrate and Sterile substrate) with the same letter (c or d) are not significantly different (*P* < 0.05) as compared within sample group (Eggs or No Eggs).

**Table 3:** Percent ± SE* black soldier fly, *Hermetia illucens*, egg deposition in oviposition sites inoculated with differing egg treatments using conspecific eggs

	Sterile†	Non-Sterile	H_2_O Rinse‡	No Eggs
	(T1)	(T2)	(C1)	(C2)
Eggs Deposited^1^	18.6 ± 1.6^a^	39.2 ± 2.4^b^	34.8 ± 2.8^b^	7.4 ± 2.9^c^

^1^Treatments with the same letter are not significantly different (*P* < 0.05: Tukey's Post-hoc Pairwise comparisons).

*Twelve replicate experiments; ^†^Eggs sterilized with Sporgon®; ^‡^Eggs rinsed once with sterile water.

**Table 4:** Percent ± SE* oviposition response of black soldier flies, *Hermetia illucens*, to concentration curve of identified bacteria isolated from various fly sources and life stages and the lesser mealworm, *Alphitobius diaperinus*

				Concentrations (cfu/ml)				Statistics	
Source	Stage	Bacteria	Gram	0	10^4^	10^6^	10^8^	ANOVA	Kruskal-Wallis
				Percent Oviposition ± SE					
CM	egg	*Hafnia* sp.	−	22.0 ± 5.5	31.9 ± 5.2	25.6 ± 4.7	20.4 ± 2.2	*P = 0.361*	*P = 0.320*
		*Ignatzschineria* sp. 1	−	17.4 ± 2.6	29.3 ± 2.1	29.9 ± 3.2	23.5 ± 6.7	*P = 0.180*	*P = 0.223*
		*Ignatzschineria* sp. 2	−	42.9 ± 0.9^a^	24.0 ± 0.9^b^	13.8 ± 1.7^c^	19.3 ± 1.9^b,c^	*P* = 0.001	*P* = 0.02
		*Ignatzschineria* sp. 3	−	49.5 ± 5.8^a^	22.4 ± 5.1^b^	12.3 ± 3.0^b^	15.7 ± 1.0^b^	*P* = 0.0009	*P* = 0.044
CR	3^rd^	*Klebsiella* sp.	−	22.6 ± 6.4	28.1 ± 3.4	25.1 ± 3.3	24.2 ± 3.4	*P = 0.841*	*P = 0.764*
	instar	*Morganella* sp.	−	21.9 ± 5.4	28.7 ± 3.8	29.7 ± 4.4	19.6 ± 5.4	*P = 0.491*	*P = 0.618*
		*Proteus* sp.	−	25.0 ± 2.8	20.8 ± 3.4	30.6 ± 2.0	23.6 ± 1.0	*P* = 0.103	*P* = 0.218
		*Providencia* sp.	−	17.2 ± 0.6^a^	19.1 ± 3.7^a^	35.6 ± 3.7^b^	28.1 ± 2.0^a,b^	*P* = 0.006	*P* = 0.044
		*Staphylococcus* sp.	−	23.6 ± 8.6	18.5 ± 7.4	22.8 ± 3.0	35.1 ± 13.1	*P* = 0.607	*P* = 0.715
HI	egg	*Bacillus* sp.	+	48.7 ± 7.9	10.3 ± 4.1	18.8 ± 3.5	22.3 ± 7.6	*P = 0.011*	*P = 0.063*
		*Empedobacter* sp.	−	19.3 ± 2.8	36.1 ± 6.3	23.6 ± 3.1	20.9 ± 2.9	*P = 0.068*	*P = 0.113*
		*Enterobacter* sp.	−	19.2 ± 0.7	25.0 ± 4.0	25.5 ± 1.0	30.3 ± 5.4	*P* = 0.065	*P* = 0.123
		*Kurthia* sp.	+	24.3 ± 4.3	25.6 ± 8.0	19.3 ± 1.1	30.1 ± 3.7	*P = 0.489*	*P = 0.392*
		Mixture		21.2 ± 9.4^a^	26.6 ± 8.2^a,c^	23.0 ± 6.5^b,c^	29.2 ± 7.7^a,c^	*P* = 0.0004	*P* = 0.02
		Constituents:							
		*Cellulomonas* sp.	+	12.0 ± 1.0	30.0 ± 19.5	16.1 ± 0.5	41.9 ± 20.0	*P* = 0.495	*P* = 0.321
		*Gordonia* sp.	+	17.6 ± 4.6^a^	31.3 ± 3.3^b^	22.9 ± 1.1^a,b^	28.3 ± 1.3^a,b^	*P* = 0.0004	*P* = 0.02
		*Microbacterium* sp.	+	17.9 ± 4.0	35.1 ± 8.3	25.0 ± 1.8	22.0 ± 3.3	*P* = 0.172	*P* = 0.340
		*Micrococcus* sp.	+	19.1 ± 5.0	29.4 ± 1.8	24.8 ± 3.1	26.7 ± 2.2	*P* = 0.396	*P* = 0.418
AD	adult	*Acinetobacter* sp.	−	36.2 ± 0.7^a^	23.2 ± 4.0^a^	21.3 ± 1.0^a^	19.3 ± 5.4^b^	*P* = 0.0009	*P* = 0.044

*Twelve replicate experiments. AD = *Alphitobius diaperinus* (Lesser Mealworm); HI = *Hermetia illucens* (Black soldier fly); CM = *Cochliomyia macellaria* (secondary screwworm); CR = *Chrysomya rufifacies* (hairy maggot blow fly).

a–cSample groups with the same letter are not significantly different (P < 0.05) as compared across concentrations.
